# Tensions in Communication between Children on Antiretroviral Therapy and Their Caregivers: A Qualitative Study in Jinja District, Uganda

**DOI:** 10.1371/journal.pone.0147119

**Published:** 2016-01-19

**Authors:** Phoebe Kajubi, Susan Reynolds Whyte, David Kyaddondo, Anne Ruhweza Katahoire

**Affiliations:** 1 Child Health and Development Centre, College of Health Sciences, Makerere University, Kampala, Uganda; 2 Department of Anthropology, University of Copenhagen, Copenhagen, Denmark; The George Washington University School of Medicine and Health Sciences, UNITED STATES

## Abstract

**Introduction:**

HIV treatment and disclosure guidelines emphasize the importance of communicating diagnosis and treatment to infected children in ways that are appropriate to children’s developmental stage and age. Minimal attention, however, has been given to communication challenges confronted by HIV-infected children and their caregivers. This study examined the tensions between children and their caregivers arising from differing perspectives regarding when and what to communicate about antiretroviral therapy (ART).

**Methods:**

This qualitative study was conducted between November 2011 and December 2012 and involved 29 HIV-infected children aged 8–17 years on ART and their caregivers. Data were collected through observations and in-depth interviews, which took place in homes, treatment centres and post-test clubs. Children and caregivers were sampled from among the 394 HIV-infected children and (their) 393 caregivers who participated in the cross-sectional survey that preceded the qualitative study. ATLAS.ti. Version 7 was used in the management of the qualitative data and in the coding of the emerging themes. The data were then analyzed using content thematic analysis.

**Results:**

While the children felt that they were mature enough to know what they were suffering and what the medications were for, the caregivers wanted to delay discussions relating to the children’s HIV diagnosis and medication until they felt that the children were mature enough to deal with the information and keep it a secret and this caused a lot of tension. The children employed different tactics including refusing to take the medicines, to find out what they were suffering from and what the medications were for. Children also had their own ideas about when, where and with whom to discuss their HIV condition, ideas that did not necessarily coincide with those of their caregivers, resulting in tensions.

**Conclusions:**

Guidelines should take into consideration differing perceptions of maturity when recommending ages at which caregivers should communicate with their children about diagnosis and ART. Health care providers should also encourage caregivers to recognize and respect children’s efforts to learn about and manage their condition. Children’s questions and expressions of feelings should be treated as openings for communication on these issues.

## Introduction

The World Health Organization (WHO) estimated that there were 3.4 million children under 15 years living with HIV globally in 2011, with sub-Saharan Africa being the home to over 90% of the pediatric HIV-infected population [1]. In Uganda, 13% of the estimated 1.6 million people living with HIV are children under 15 years [2]. Expanded access to antiretroviral therapy (ART) increased the number of HIV-infected children receiving treatment from 35,443 in 2012 to 43,803 in 2013 [2]. Given the fact that most children are infected perinatally, many are introduced to ARV drugs while very young and unable to comprehend the gravity of their diagnosis [3, 4]. As the children grow older however, the necessity for open communication about their diagnosis and treatment becomes more urgent in order to ensure their understanding and continued adherence to the medication [5].

Literature on disclosure of pediatric HIV infection notes that the child’s age is an important predictor of whether or not disclosure occurs and is also key to how much information is communicated to the children regarding their diagnosis and ART [6–11]. A common observation from studies on disclosure and disclosure guidelines is that communication with HIV-infected children about their diagnosis and ART should be regarded as a process that takes into account the cognitive development (developmental needs, psychosocial maturity and understanding) of the child, regardless of their chronological age [4–6, 9, 12–17]. This however poses a challenge for caregivers [14, 18–20], especially since pediatric ART involves a broad age range of children. It requires caregivers to adapt their communication with HIV-infected children to what they perceive as the children’s developmental needs and understanding [5, 13]. This does not always coincide with the children’s own perception of their needs, resulting in tensions between caregivers and their children.

Unlike other chronic illnesses such as cancer, communication about perinatally acquired HIV infection is often complicated by the stigma associated with the disease, the likelihood that the parents are also infected or died from the disease, the need to conceal this information and having to live on lifelong medication [8, 9, 13, 14, 18, 21]. As a result, caregivers of HIV-infected children are often reluctant to discuss their illness and ART with them, disregarding children’s capacities for understanding. Caregivers’ postponement of disclosure may relate more to their own readiness to deal with the disease than to the child’s capacity to understand [19, 20, 22, 23]. Guidelines on HIV Counselling and Testing for Adolescents note that when caregivers have not informed children about their HIV status, it may be because they are concerned about their own HIV status and are unwilling to confront the fact of their responsibilities in passing on the infection to the children [24].

Furthermore, disclosure models often do not acknowledge that disease understanding is a gradual lifelong process and thus fail to take into consideration the needs of younger children [13]. Other studies suggest that children’s understanding of their disease does not start at a particular age but becomes more sophisticated with age [13, 25]. So whilst communicating with HIV-infected children about their diagnosis and ART is crucial to long-term disease management, it remains a challenge to caregivers [26, 27].

In low-resource settings such as Uganda, standardised, culturally appropriate guidelines and resources to facilitate the communication process are lacking or limited [9, 12, 26, 28, 29]. Previous research has tended to focus more on providers’ and caregivers’ concerns and perspectives regarding disclosure [6, 8, 13, 17, 19, 30], whilst minimal attention has been given to HIV- infected children’s’ perceptions regarding communication about their health and ART. Furthermore, minimal attention has been given to the communication challenges arising from differences in perceptions of children and caregivers regarding the children’s ability to understand and deal with their diagnosis and treatment. A systematic review of 32 studies on disclosure of HIV status to children in resource-limited settings argued that while organizations such as WHO have published recommendations for disclosure of HIV status to children, the recommendations lack a broad evidence base and do not take into account different cultural views on age, maturity and psychosocial development [9].

In light of the above, this study explored communication between caregivers and children on ART and examined the underlying tensions arising from differing perspectives regarding when and what to communicate about ART.

## Methods

### Study site

The study was conducted in Jinja District, located on the shores of Lake Victoria in Eastern Uganda. Jinja District has a population of more than 470,000 people, of whom 79.1% live in rural areas [31]. The district has a very youthful dependent population; with slightly more than half (56%) below 18 years. A tenth (11%) of these children are orphans, defined in Uganda as a child who has lost one or both parents and is below the age of 18 [32]. The main economic activities in the district are subsistence farming, fishing and trading, with most of the households depending on earned income [33]. The district has a total of 69 health facilities comprising 49 Government (public), 17 NGO and 3 Institutional (Army, Police and Prisons) health units. At the time of the study, 6 Government and 3 private health facilities were providing ART for children. The overall HIV prevalence in the district was estimated at 5.8% in 2011, lower than the national prevalence rate of 7.3% [34].

### Study design, participants and sampling

A cross-sectional survey, which is fully reported elsewhere [35], served as the foundation for the qualitative study described here. The survey involved 394 HIV-infected children aged 8–17 years on ART and their (393) caregivers. It was conducted between September and December 2011 in nine health facilities providing ART in Jinja District, Uganda. A proportionate number of HIV-infected children selected from each health facility was determined by probability proportional to size of the facility population of infected children. Children from each facility were randomly selected using systematic sampling. Children were considered eligible if they were aged 8–17 years, registered in the health facilities, were on HIV medicines and were accompanied by their primary caregivers on the day of the interview. Children did not have to know their HIV status. A primary caregiver was considered to be the person who lived with the child (including but not limited to biological parents), participated in the child’s daily care, and was the most knowledgeable about the child’s health and medicines. The primary caregivers were identified and screened for eligibility during the survey with the help of the paediatric counsellors.

A structured interviewer-administered questionnaire captured children’s socio-demographic characteristics and communication practices (frequency and content) with their caregivers regarding their medicines. For caregivers, a separate interviewer-administered questionnaire was administered to elicit demographic characteristics and communication practices with children concerning the medicines. The caregiver interview contained some open-ended questions upon which this article draws: if they explained what the medicines were for; whether they had informed the children what they were suffering from; if not, why not and when they intended to do so.

The qualitative study was conducted between November 2011 and December 2012. The 29 HIV-infected children who participated in the qualitative study were identified during the survey with the help of health care providers. They were purposively sampled (using non-probability sampling) to include HIV-infected children of different ages, sex and family situations (residence including the homeless; orphan status (double, maternal and single orphans); caregiver dynamics e.g. biological parents, fathers only, mothers only, foster parents, grandparents, maternal /paternal relatives, non-relatives), education levels, membership/non-membership to a post-test club and disclosure status. Purposive sampling that involves non-probability samples entails choosing cases on purpose, not randomly, appropriate for in-depth research on sensitive topics that can take months of participant observation fieldwork [36]. The sample size was large enough to enable exploration of a diverse range of children’s experiences, perspectives and communication practices, and small enough to be feasible for one researcher to follow up.

### Data collection methods

In-depth interviews with children and observations were the main methods of data collection used in this study. The first author (PK) lived in the study area during the 12 months of data collection. During this time she interacted with children, their caregivers and other significant people in their families and collected data on their daily lives including their illness and treatment experiences.

#### Observation

Observations were done simultaneously with the interviews. During visits to the children’s homes, observations of children’s interactions with family members were conducted. The first author listened to their conversations and observed their communication practices (what children and family members talked about, who children talked to most of the time, how they interacted, their facial expressions and body language). During conversations with the children and their caregivers, PK would interject to probe for clarification about issues that arose and were considered to be pertinent. This helped to build rapport with children that was conducive for in-depth interviews that followed after repeated visits and interactions. Bernard (2006) explains that informal interviewing and or unstructured interviewing are a good method to be used at the beginning of participant observation fieldwork and are excellent for building initial rapport with people before moving to more formal interviews [36]. The observations conducted after the in-depth interviews shed light on some of the information obtained from the interviews.

The time spent and visits made to the homes of each of the children ranged between one and twelve times, depending on the perceived maturity of the child, whether the child’s HIV status had been disclosed to them and relationship of the child to the caregiver. For the younger children (8–10 years) who had not been disclosed to and in homes where there was no open communication about the child’s illness and medicines, only one or two visits were made, even then, caregivers controlled access and conversations with the children for fear of inadvertent disclosure. Time spent in these homes ranged between three to four hours per visit. Interactions with such children and their caregivers were more at the treatment centres during the monthly clinic appointments. For the older children (13–14 year-olds) who had been disclosed to, more frequent visits were made to these homes and the time spent was longer, ranging from five to six hours at a time. Caregivers allowed the researcher to spend time alone with children and even to take walks with them. Older children preferred meeting at treatment centres, club gatherings and other public settings. More time was spent with them, ranging from six to eight hours depending on the duration of the activities they were involved in.

Subsequent visits and interactions added cumulatively new perspectives on children’s everyday practices and experiences in different spaces and how these other situations influenced their communication practices and understanding of their medicines and health. New versions of their stories emerged as months went by and as the researcher (PK) got to know them better, yielding better understandings of changes in their life situations over time.

The first author maintained a daily notebook in which each day’s activities were recorded; field notes written during visits were expanded at the end of each day.

#### Interviews

The in-depth interviews were conducted after a period of repeated visits in the homes and interactions with the children in other social spaces including treatment centres and club gatherings during which time rapport was established with the children and caregivers. A pre-tested interview guide was used to conduct individual in-depth interviews with the children. Interviews were conducted with older children who were already aware of their HIV diagnosis (21/29) and these mostly included 13–17 year olds. Children who were perceived to be young/ immature and who did not know their HIV status were not interviewed because their caregivers feared inadvertent disclosure.

Due to the sensitive nature of the subject, the interviews started off as conversation on issues of interest to the children, including their social lives (home, school, holidays, friends, relatives, and daily activities) and progressed to questions about their family and friends and whom they confided in when they had good news or bad news. Eventually the conversations focused on their medicines and what they were told about them and whom they discussed them with in case they had any questions. The questions were deliberately phrased in a way that no mention was made about HIV/AIDS or ART unless the children themselves brought them up. Table 1 below shows a summary of the topics that were included in the in-depth interview guide.

**Table 1:** Summary of the topics in the in-depth interview guide.

TOPICS	SUB-TOPICS
**Experiences considered most important in the life of the child**	Schooling/lack of schooling experiences; a typical week of schooling; vacation/holiday-what they do, who they visit; involvement in social events e.g. sports, drama, post-test clubs, relations with peers; whom they confide in when they have good or bad news.
**Socio-demographic information**	Age, birth/parents, education status, residence i.e. who the child lives with/family relations, relationship to caregiver, employment of caregiver, living conditions, number of siblings.
**Health and medicine experiences**	Why and how often they go to the treatment centres; who escorts them; what takes place when they go; what medicines they are given and how often they take them; how long they have been taking the medicines; where they keep the medicines; who helps them to take the medicine; who they talk to about the medicine at home, school, neighbourhood; what they talk about; who else at home takes similar medicines; what they understand by the need to be on lifelong/daily medication
**Learning about status/experiences of disclosure**	Reasons they had been given for taking daily medicines; who told them; their experiences and reactions when they learnt reasons for taking daily medicines; how they came to know the illness/health condition for which they took daily medicines; who told them; what exactly they were told; how they were told; age at which they were told; their experiences/reactions when they were told about their illness; what they understand by having illness.
**Communication about illness and treatment in different social spaces**	People who knew about their illness/health and medicines at home, school, in the neighborhood, and how they came to know; people they had told about their health and medicines; reasons for telling such people; people’s reactions when they were told; who they normally communicated with about their illness and treatment and where; who they think deserved to know about their illness and why; what they liked/disliked to hear about their health and medicines; questions/challenges of being on daily medicines; whom they talk to about these challenges; how they could be supported to live on daily medicines.

The children mostly spoke Luganda during the interviews and conversations; a few mixed it with English and Lusoga. The first author (PK), who conducted the interviews, was conversant with the both languages (Luganda and Lusoga) so no interpreter was needed. The interviews lasted between 40–45 minutes and were audio recorded with participants’ permission.

### Data management and analysis

Transcripts of in-depth interviews and detailed field notes taken during conversations and observations formed the data set. Two graduate research assistants (a male and female) experienced in qualitative research and conversant with the local languages used during the interviews, transcribed and translated the recorded interviews into English. Transcripts were cross-checked and verified by one of the research assistants and the first author (PK) for consistency and accuracy.

Data were analyzed using content thematic analysis. Preliminary data analysis occurred concurrently with data collection during quarterly de-briefing meetings PK had with the co-authors. Discussions during these meetings helped to identify emerging issues and also informed further data collection. Emerging themes were also identified during the process of typing and reading through the transcripts and detailed field notes. This was an important part of preliminary analysis and identification of thematic categories. It facilitated familiarisation with the data. The overall research objectives and topics in the interview guide also informed the process of developing a codebook. PK and one of the research assistants generated a list of themes during the process and this formed a framework for a codebook, which was discussed with the co-authors. Codes were progressively developed, reviewed by the co-authors and applied to the transcripts. The major themes in the codebook that are pertinent to the paper included: children’s communication practices about their health and medicines (who they talk to, what they about), age-related differences and challenges in communication; experiences and process of understanding of their health/illness and medicines; questions, dilemmas, challenges and concerns of being on daily medicines and who they talked to about these; challenges encountered in understanding and communicating about their health and medicines; what they liked/disliked to hear about their health and medicines and suggestions on how they could be supported to live on daily medicines.

The compiled data set was eventually imported into Atlas.Ti.version 7 (Atlas.Ti.Scientific Software Development, Berlin Germany), which was used to more systematically manage, code and retrieve data. PK read through the imported data set, line by line, identifying segments of the data that related to the themes in the codebook and assigned them relevant codes. During this process of systematic coding, more themes and sub-themes were identified, discussed with the co-authors and also assigned relevant codes. In addition ‘memos’ that contained thoughts about the selected segments of highlighted and coded data were written. Together the codes and memos formed the basis for the thematic analysis where patterns and meanings were established. During the coding process, quotations illustrative of children’s and caregivers’ communication practices, perspectives and experiences were identified and used in the presentation of findings. Through this process the large data set was condensed enabling the identification of recurring themes, linkages and similarities. On the basis of this, impressions were developed and eventually explanations. All co-authors provided valuable input during analysis and interpretation of results in form of complimenting and contesting understanding of study results.

### Ethical Considerations

This study was approved by Makerere University College of Health Sciences, Higher Degrees, Research and Ethics Committee and the Uganda National Council for Science and Technology. At lower levels, permission was also obtained from Jinja District Administration and the treatment centres where children on ART included in the study were accessed. Informed written consent was obtained from caregivers for their own and children’s participation in the study. Written assent was also obtained from children whose caregivers had provided consent. During the consenting and assenting process, the purpose of the research was explained to the caregivers and their children as a study of children’s understanding and communication about their health and medicines. Only children 8 years and older were enrolled in the study, since according to Ugandan ethical guidelines that is the age of assent. Pseudonyms are used for the children to ensure confidentiality.

### Analytical concepts

The article draws on the analytical concepts of ‘childhood’ and ‘children’s agency’ in analyzing children’s communication with their caregivers about their diagnosis and being on ART. We adopt an approach that considers age/development as culturally defined; but we show that there is not always consensus about the markers of maturity. Drawing on the concept of children’s agency, this paper analyses the tensions that exist between children and their caregivers, as a result of their differing perceptions of maturity. Children’s agency, in this case, is defined as their capacity to act and shape the circumstances in which they live [37], and their ability to reflect and act independently, thus challenging their social environments [38, 39].

Both the UN Convention on the Rights of the Child (CRC, United Nations 1989, Article 1) and the Constitution of the Republic of Uganda (1995) define a child as a person below the age of 18 years [40, 41]. However, anthropological studies show that there are varying views concerning the concept and duration of childhood [37, 42–48]. Evidence of cross-cultural variations in childhood is documented extensively in literature and differences have been observed in constructions of childhood between the western and the developing world [49]. Meinert (2009) observed that among the Iteso people in Eastern Uganda, age in years is seldom used as a marker of maturity; the definition of a child depends on their life situation as well as the specific context [50]. Among the Iteso, a 17 year old female is not considered to be a child in the home into which she has married and given birth. However, when she goes to visit her parental home, she is considered a child. Similarly, a 25-year-old son, who still lives at home, has not yet married, and has not yet built his own house is, on the contrary, considered a child in some aspects [50]. A study conducted among the Baganda in central Uganda suggested that childhood is constructed or institutionalised rather than biologically determined because of the differences in experiences in children’s lives even within the same family and locality [42]. Childhood is described by anthropologists as a highly diverse life phase shaped not simply by biological or psychological universals but also, by personal and environmental factors [51]. Bluebond-Langner et al. suggest that as we study children and childhoods, we need to confront social reality and continue to problematize the nature and development of the child as an individual [52]. This line of thinking renders children as agents of their own development who, even during times of great adversity, consciously act upon and influence the environments in which they live [51]. However, proponents of the concept of ‘agency’ argue that children’s ability to exercise agency is heavily limited by the economic factors that threaten their survival, profoundly constrained by social contexts [37] and intertwined cultural and relational practices that silence their voices in their families [50, 53] and in the national and international contexts that contextualize efforts to improve their health [38, 39]. They argue against a tendency to view agency as a child’s ability to engage in any form of independent action per se and suggest the need to pay attention to the extent to which children have access to the resources they would need to act in ways that take them closer to the lives they themselves would want to lead [38].

Against this background, discussions on the definitions of *child*, *youth*, *and childhood* and concepts of *age*, *agency*, *development roles*, *and responsibilities* continue [52]. Our interest in the concept of childhood and the anthropological work on different stages and duration of childhood is to show that whereas chronological age is relevant and very convenient for policy makers to use in the development of guidelines, in reality caregivers of children in Uganda do not rely exclusively on age, but use local concepts or their own perceptions of maturity/development in determining how and when to communicate with HIV-infected children about their diagnosis and treatment. These concepts may or may not coincide with the children’s chronological age. Moreover, the children themselves may not share their caregivers’ views about their maturity.

## Results

### Description of participants

Of the 394 HIV-infected children interviewed during the survey, 218 (55.3%) were females and 295 (74.9%) were below 15 years. The mean age was 12 (SD 2.7). Slightly over half, 206 (52.3%) of the children lived with caregivers other than their biological parents. Majority, 267 (67.8%) were orphans comprising, 144 (53.9%) single and 123 (46.1%) double orphans. Almost all, 365 (92.6%) children were attending school.

Of the 393 caregivers of HIV-infected children who participated in the survey, the majority (80.9%) were female. Their mean age was 40 (SD 11.4). The majority (78.4%) had been to school and about half (50.3%) had attained secondary education and above. The most common occupation (41.5%) was subsistence farming.

Participants in the qualitative study comprised 29 HIV-infected children aged between 8 and 17 years. Their mean age was 12 years and that of caregivers 40 years. Children’s median age was 12 years, (IQR, 10–15) and range was 8–17 years; while that of the caregivers was 39 years (IQR, 32–46) with a range of 15–80 years. Over half of the children (16/29) and majority of the caregivers (21/29) were female. The average number of siblings was four, with some having none. Most children were in school, the majority (17/29) being in primary; a few (7/29) had dropped out of school.

Majority (21/29) of the children were single or double orphans; only five had both parents alive. Slightly over half of them lived with a caregiver other than their biological parent, commonly maternal grandmothers. Children’s caregivers were mostly small-scale farmers.

### Caregivers’ perspectives regarding disclosure

Findings from the survey of the 394 HIV-infected children and their caregivers [35] revealed that only half of the children had been told they were taking medicines for HIV. The other half, who largely comprised 8–10 year olds, were told they were taking medicines for other diseases or were not given any explanation at all. Caregivers of children in this category were asked when they intended to tell the children what they were suffering from and why they were taking medicines. This data is drawn from the caregivers’ responses to the open-ended questions in the survey. Whether or not they mentioned chronological age, caregivers pointed out markers of maturity on which they based their decisions regarding whether, when and what to communicate to children about their medicines.

Findings from the survey showed that the *majority* of caregivers suggested that they would communicate to the children reasons for taking daily medicines when they are *mature enough to understand* the diagnosis and to *keep it a secret* as illustrated below:

....*When she turns about 12 years*, *I will disclose to her because she will be understanding and can keep this a secret*.

(Mother of 8 year old girl).

.....*I will tell her when she turns 15 years because she will be able to understand and she won’t be telling others what she is suffering from*.

(Grandmother of 10 year old girl).

The caregivers said the children needed to be old enough to understand the need for discretion, which they considered a marker of maturity. Caregivers also based their decisions on biological markers of physical maturity as reported below:

.....*I am about to tell her because she is now a big girl who can get tempted to sleep with men*.

(Maternal aunt of 12 year old girl).

.....*I am planning to do so very soon because she is about to get her periods and she has to be cautious so I intend to ask the health care providers on how to do it*.

(Mother of 12 year old girl)

Other important markers of the child’s level of maturity included: responsibility for self-care, and child’s emotional ability to handle negative circumstances (not worrying) as shown below.

....*When she reaches primary five*, *at that age she will be understanding and she can be responsible to care for herself*, *keep the medicines and take drugs by herself*.

(Paternal aunt of 9 year old girl).

.......*When he joins secondary school*, *that’s the time when he will be able to understand the circumstances and he can even care for himself*.

(Grandmother of 10 year old boy)

....*I intend to tell her when she turns 15 years because at that age she will be understanding and will not worry*. *I do not want to tell her now her now because she will get worried*.

(Grandmother of 10 year old girl)

We noted that the markers of maturity mentioned underscore caregivers concerns about whether the children are old enough to comprehend that the disease they have is incurable and that they have to remain on medications for life. The children have to understand that the disease is socially discrediting for them and their families. It is sexually transmitted so it is not just the fear that the children will become pregnant or infected with STIs; it is also the concern that they could infect others and the responsibility this carries. Caregivers are thus worried about not only the physical wellbeing of the children but also about their emotional well-being, how the children are likely to interpret the information about their diagnosis.

### Communicating with children on ART

The qualitative study revealed the challenges of caregivers’ communication with children of different levels of maturity. The age range 8–17 years was conceptualized by caregivers as a spectrum of children who were seen as: still young; children who perceived themselves to be mature but were perceived by their caregivers to be immature; and those who perceived themselves to be mature and were also perceived by their caregivers as mature. Three corresponding patterns of communication were observed in the interaction between HIV- infected children and their caregivers. Among children perceived to be still young/immature, communication about their health and medicines was dominated by their caregivers, although curious children tried to push for more information. Among children who perceived themselves to be mature but were perceived by their caregivers to be immature, the communication was characterised by tension with more children asking questions about their health and medicines and expressing discontent with some of the responses. The communication involving those perceived to be mature was dominated by the children asserting themselves and evincing an increasing desire to be more independent.

### Dependence and curiosity: ‘*Why do I have to take medicines*?’

Children perceived by caregivers as ‘still young’ or immature in most cases did not know their diagnosis as illustrated below:

*I have deliberately kept this a secret and have not disclosed to them*, *they are too young*. *In the beginning when Derrick was tested for HIV*, *he cried a lot and he said he feared that they were going to tell him that he has slim*. *I told him*, *‘That’s impossible*, *a young child like you cannot have slim”*, *even the nurse told him that you are too young to have slim’*.

(Grandmother of Derrick age 10 and Yusuf age 9)

During visits to their homes, it was observed that their caregivers kept their medicines for them, reminded them to take them and observed them as they took the medication. Observations at the treatment centres also revealed that caregivers accompanied the younger children to the treatment centres for check-ups and medicine refills. The children were not all necessarily young in terms of age, but were treated by their caregivers as such. Observations also revealed that the caregivers protected the children perceived to be still young from inadvertent disclosure by controlling their interactions with health care providers at treatment centres. Caregivers spoke on their behalf during clinic visits and also during the researcher’s visits to their homes, even when conversations and/or questions were directed to the children.

However, despite caregivers’ withholding information from the children about their diagnosis, the children were curious about the medicines and why they kept taking them even when they were well and asked their caregivers these questions. This was true for all the younger children followed up as part of this study whose ages ranged from 8 to 10. Derrick (age 10) and his brother Yusuf (age 9) persistently asked their caregiver (grandmother) questions such as:

*why am I taking medicines*, *what are the medicines for*, *when will I stop taking medicines*, *what am I suffering from*?

During one of the visits to the home of Afisa (age 10) and Jamillah (age 9), their mother explained that she had not planned to disclose to her young children but was forced by their continuous nagging and persistent questioning. She explained that when people in their community found out that they were infected with AIDS, Afisa began to be taunted by the friends she played with, who told her, ‘*you are suffering from slim [AIDS]’*. At the time Afisa did not know what it meant, but she told her mother, who at first was reluctant to explain. However, due to persistent questioning by Afisa, she eventually told her children the truth that they were HIV-positive but, cautioned them never to tell anybody. She believed that the children’s curiosity was satisfied because they never asked her any questions again, even though they did not understand what the diagnosis meant.

Deception and half-truths regarding reasons for taking daily medicines and repeated visits to treatment centres were common. It was observed that caregivers used co-existing medical conditions as explanations in response to children’s persistent curiosity; they thought children hardly understood anything about their illness even when they did. This was a source of tension. Farouk’s grandmother, who perceived him as being too young at nine years to be informed he had AIDS, repeatedly told him that he was taking medications for persistent malaria. She confided in PK that she had exhausted all possible lies to convince her grandson to take the medicines and to come to the treatment centre monthly for ARVs. She was still adamant about not wanting to disclose to Farouk and wanted advice on what to tell him in order to convince him to take the medicines and to pick up his refills. On the particular clinic visit when PK met his grandmother, Farouk had initially refused to come arguing that he was not sick. So the grandmother pretended she was suffering from a headache and convinced him to accompany her to the treatment centre. It was observed at the clinic that Farouk took responsibility for obtaining his grandmother’s medications, but did not realize that they were his own. Farouk later asked PK why he had persistent stomach pains and a recurring skin rash yet he took medicines daily.

Caregivers believed that due to immaturity, the children would not be able to keep their HIV status a secret even if they were told. Flavia’s aunt concealed her HIV status from her for that reason. However, she disclosed to Flavia’s sister (age 13), who took similar medicines and was perceived to be more mature. The sister chose to disclose to Flavia, informing her that they were both infected with HIV and were taking medicine for AIDS. Flavia (age 8) immediately asked their aunt whether it was true and her aunt denied knowledge of it. Flavia nevertheless told her friends with whom she played in the neighbourhood. When her aunt got to know that there was a rumour going around in the neighbourhood that they were infected with HIV, she was disturbed and cautioned Flavia whom she thought did not understand anything. This she confirmed when she overheard her explaining to one of the household members who was cutting banana leaves that she should not use the same knife on two banana trees because the one infected with AIDS would infect the other banana tree. It was then that her aunt concluded that Flavia was still too young to understand the meaning and seriousness of their diagnosis. Caregivers of children perceived to be young silenced children’s questioning about their health and limited the communication to taking medicines.

### Confrontations and demands to know: *‘If you don’t tell me…*.*’*

Children who perceived themselves to be mature and ready for communication about their health and medicines were better able to voice their experiences and concerns as observed below:

*Matthew is very young*, *only 11 years old*, *but he behaves and thinks like an adult*, *he may seem to be young but he seems to be mature in his understanding*, *sure of what he is talking about and he has been very consistent in his conversation about issues concerning his life*. *He desperately wants to find his mother to ask her why he is taking medicines and whether she and her young sister are taking similar medicines*.

(Grandmother of Matthew-11 years)

Interactions with caregivers and interviews with these children revealed that they were more assertive and confrontational in their demands to have responses to their questions to the extent that some began refusing to take medicines if their caregivers did not respond to their questions about why they were taking medicines daily and what they were suffering from. Shakirah (age 14) recounted:

*I always asked my mother when I was still young why I take the medicine and she refused to tell me*. *I insisted and I threatened her that I wasn’t going to take the medicine until she told me why*. *Then she told me*.

Matthew (age 11) persistently questioned his grandmother about the cause of his father’s death and why he had to take medicines daily. He wanted to hear the truth from his grandmother because friends at his primary school had told him that he was suffering from *slim*. During the interview, Matthew explained that he did not trust his grandmother and counselors anymore, because they had refused to tell him what he was suffering from and the cause of his father’s death. Matthew desperately wanted to find his mother (who had abandoned him after his father’s death) to know the truth.

Stella (age 14) was always sickly yet she was taking her medicines. Her mother reported:

*Stella used to ask me many questions that made me uncomfortable and worried …*.*questions such as why is it that it’s only her who is always falling sick when her siblings are ok and yet they don’t take any medicines*. *The health workers advised me to disclose to her*. *I told them that they should do it because I couldn’t…*. *Stella became too difficult to handle*. *I tried to encourage her by telling her that the 17 -year old boy at home is also on medicines for life because he is asthmatic*. *I told her many people she sees walking are also on medicines*.

In an interview, Stella confided that even though she loved her mother and appreciated the love and care shown to her, she never believed the explanations her mother gave her for taking daily medicines:

*My mother told me that I shouldn’t worry*, *that many people take the medicine but in secrecy…*. *I stopped asking her why I take the medicine*. *But I did not believe her…*. *I always doubted it because I have never seen them taking the medicine*. *I wanted to see them at least taking the medicine*.

(Stella, 14 years).

The above expressions from children suggest that they perceived caregivers’ protection as deception and betrayal. The vague explanations to their questions, the prolonged silence about their diagnosis by their caregivers conveyed an unspoken message and raised more suspicions.

Consequently, some of the children sought alternative sources of information.

Stella confronted the doctor and told her that she was fed up with the medicines. She threatened that if she was not told why she was on daily medication she would stop taking the drugs, and the doctor disclosed to her that she had AIDS. Matthew learnt of his diagnosis when he went to stay in the children’s home where HIV-infected children in very poor health condition were staying for rehabilitation.

*It’s the Sister (caretaker) who told me that I have AIDS and I could see the children in the Home*. *Some were really sick; they had skin rashes*, *and they looked as if they had kwashiorkor…I thought maybe the Sister was lying so I decided to go ahead and ask my grandmother*.

There were cases where children who believed the deceptive explanations from their caregivers expressed confusion. Samalie, who was suffering from a debilitating skin rash, reported that she was told that she was suffering from syphilis. She now wanted to know why health workers forced her to take ‘RVs’ and yet she was suffering from syphilis. Samalie expressed this confusion in the question she asked during one of the visits to her home:

*Isn’t it very dangerous to one’s health to take ‘RVs’ if one is not infected with the virus*? *I was started on HIV medicines when I am not infected*. *I was told that I was bewitched and that’s why I am suffering from syphilis not HIV/AIDS*……..*People infected with the virus have skin rashes all over their bodies*, *but for me the rashes are only on the arms and on the legs*!

(Samalie, 15 years).

These children understood that there was something wrong and that their caregivers were not being very truthful with them and were frustrated because they needed responses to their questions.

### Older Children seeking independence: *‘What does s/he know that I don’t know*?’

The children who perceived themselves and were perceived by their caregivers as being mature were aware of their diagnosis. During the interviews, they reported that they were in charge of their medicines and knew how and when to take them without being reminded or instructed as noted below:

*Me*, *myself*, *nobody reminds me to take my medicines*. *I can never run out of medicines*, *no*, *it can never happen because I always keep my appointments to come and get medicines*. *I have a small case at home where I keep my medicine and lock it up*. *No one is supposed to touch it because it is mine*. *I have the phone alarm*. *It can remind me to take my drugs*. *I only talk to the doctors*. *I don’t talk to the people at home about my medicines*. *I am independent*.

(Sheila, 17 years)

They hardly communicated with their caregivers or other family members about their health and medicines and in most cases, their caregivers did not inquire. For example, Nick (age 17), whose father and stepmother were on similar medicines, reported that he did not talk to them about his medicines, but instead preferred to consult health workers and counsellors whom he perceived as being more knowledgeable about such things. However, Nick, who habitually forgot to take his medicines died after four months of being followed up, suggesting that had he probably communicated with his caregivers who were on similar medication, he would have lived.

Conversations about health and medicines initiated by these more mature children had to do with informing the caregivers about when to pick up the medicines from treatment centres and informing them when they were unwell. The older children also reported that there were no benefits in informing school authorities about their health and medicines even when they needed to seek permission to keep appointments at treatment centres. In contrast, caregivers of the less mature children informed teachers about their diagnosis so that they would be exempted from harsh punishment and strenuous manual tasks, and granted permission to absent themselves from school to keep clinic appointments. The older ones felt that none of the teachers and other students needed to know about their health and medicines nor did they see the need for special treatment and favours at school because of their condition.

According to the older children, they were interested in people with whom they could discuss issues that pertained to them as adolescents such as sexual relationships, safe sex, prevention of pregnancies, abortion, having children, hygiene, and positive living. This was evident during the education sessions held on adolescent clinic days and post-test club activities observed by the researcher, where they were given a chance to select topics for discussion and where they were selected to facilitate sessions. They valued the chance to share time with people like themselves, who had similar experiences with medicines and challenges at home as shown from an interview with a 17 year old boy:

*When I am at the treatment centre with my fellow youths*, *the interactions are good; it’s one of the most interesting experiences in life*. *We know that we all have AIDS*. *We have to wait for all our friends to get the medicine so that we can walk back home together*. *We talk about other things*, *just the common jazz*, *music*, *favourite artists*, *talking about funny things and we laugh*. *We do not always talk about the disease*. *After we have been taught we walk up to town chatting and go back home in big groups*.

(Nick, 17 years).

The more mature children disliked threats and warnings that they would die if they did not take their medicines. During one of the visits at Stella’s home, she said quietly:

*It’s like as if death is hiding behind the door or is around the corner ready to grab you*, *which is so scary*. *I don’t want them to scare us that the disease is terrible and kills because I lose hope that if I miss taking the medicine I will die*. *I do not want to die now; I still like to see my future*.

(Stella, 14 years).

Children also mentioned that they disliked being told that they were suffering from AIDS. They felt that it indirectly implied that they contracted the disease through promiscuity and yet they were infected perinatally.

When asked about what they would like to be told about their medicines and illness, children made various suggestions ranging from not wanting to be told anything to wanting encouraging messages. Sheila (age 17) responded, ‘*Nothing*…*I don’t need anything from them*.’ Other children mentioned that they wanted encouraging messages. Gladys explained:

*They should strengthen me and tell me that I should be strong*, *that the disease is common*. *When I am ok*, *then they should say good things about me that the CD4s have gone up*, *that I look healthy and if I am not well then they should tell me to continue taking the medicines*.

(Gladys, 14years)

Betty reported that she did not want people to talk about her illness because they would discredit her. She felt that, apart from gossiping about her, they had nothing to say as shown below:

*It’s not that I don’t want them to say anything but it’s just that they have nothing to say*. *I don’t want them to gossip about me*. *People like the neighbours and their friends will hate me and also gossip about me if they get to know that I have AIDS*. *They will say that I have AIDS and that people shouldn’t associate with me*.

(Betty, age 13)

Other children reported that the mere mention of their being infected with AIDS, automatically translates into their being ostracized both openly and subtly.

While the more mature children had been disclosed to and perceived themselves as being in charge of their lives and of their medicines, those less mature manifested more ‘self-stigma’, which they expressed in different ways. To use Goffman’s terminology [54], they adopted the view of ‘normals’ that they were discreditable. Not wanting to talk about their health and not wanting others to do so because they expected discrimination potentially undermined the support that they could get. Their fear of being discredited was not communicated and left them more vulnerable.

## Discussion

This study uncovered the complexities of communicating diagnosis and treatment with infected children of different chronological age. Caregivers’ perceptions of the children’s immaturity did not generally fit with international guidelines that advise informing children of their diagnosis when they are aged 6–12 years) [5, 12]. They were more guided by their perceptions of the child’s developmental stage, a consideration also recognized in most guidelines and in existing studies [4–6, 12–17]. Our data showed that the ability to understand the diagnosis, without worrying about it, and being able to keep it a secret, were important markers of maturity and social competence perceived by most caregivers as indicators of the child’s readiness to be involved in discussions about their HIV diagnosis and treatment. This finding is consistent with other studies on disclosure to HIV-infected children in resource- limited settings [9, 13, 18, 55, 56]. Caregivers’ concern with children’s ability to maintain discretion is indicative of the social stigma still attached to HIV.

Other markers of maturity noted by caregivers included: physical maturity/biological markers (showing signs of being attracted to the opposite sex and onset of menstruation), responsibility for self-care (children’s ability to keep their medicines, remembering to take medicines without being reminded) and emotional maturity or stability.

A central contribution of our study was to focus on children’s agency in difficult conditions, allowing us to uncover the tension between caregivers’ perceptions of developmental stage and children’s own perceptions of their maturity and agency. Skovdal and Daniel noted that literature on HIV continues to be largely unmindful of children’s agency and the fact that children in many parts of rural sub-Saharan Africa are trained from an early age in a variety of life skills to prepare them for life’s struggles [39].

Our data showed that across all age groups communication between children and caregivers was constrained. Caregivers were in a dilemma regarding whether, when and how much to communicate to children about their diagnosis and treatment and tended to withhold information even as children grew in curiosity, competence and desire for independence. In the face of caregivers’ protective stance, children challenged adult ideas of what they should know. A major strength of our study is the involvement of children of different ages and exploration of their differing perspectives on communication regarding their health and medicines. While other studies point to some of the issues of deception and over-protection on the part of caregivers [20, 29, 57, 58], few have consistently worked with children [17, 57]. Our emphasis on children’s perspectives follows from our analytical concern with children’s agency.

Although the younger children were kept in ignorance about their diagnosis, they showed curiosity and posed questions to their caregivers about the daily medicines they were instructed to take and the repeated visits to treatment centres, similar to what is reported by other studies [13, 15, 59]. Children’s persistence put pressure on caregivers to adopt various strategies in responding to children’s questions, which included deception or explanations related to other medical conditions. Caregivers considered them ‘too young’ to involve in discussions regarding their diagnosis and treatment. Previous studies identified a similar phenomenon where partial disclosure, pretense, denial, concealment or complete silence are common strategies adopted by caregivers when they feel unprepared for full disclosure [11, 20, 26, 28, 58]. A recent study in Western Uganda that explored caregiver perceptions and motivation for disclosing or concealing the diagnosis of HIV infection to children on ART found that almost all caregivers indicated that they had told a lie when children confronted them with questions about their health status [58]. Another study in the neighboring Democratic Republic of the Congo also found that caregivers’ communication was intentionally misleading or ambiguous with an intention to draw a child away from considering HIV as a possible diagnosis [11].

The findings of our research support those of previous studies, which recommend the need for professional support to caregivers throughout the process of communicating developmentally appropriate information to HIV-infected children about their health and medicines [10, 11, 26, 59]. One study in particular suggests that health care providers could assist caregivers in developing appropriate responses to children’s questions about their health as they grow older instead of deflecting information [11].

Tensions between caregivers and children were more pronounced among children who perceived themselves as mature despite the contrary perception of the caregivers; such relations were characterised by children’s more intense inquiry, suspicion, demands and threats. The finding that caregivers were still protective of them and were reluctant to involve them in discussions regarding their health and medicines agrees with another study that found that despite their parents’ stance of protective communication, children were aware of their illness and impending death [60]. Children in our study saw protection through the concealment of information as outright deception and betrayal of trust and exerted agency by seeking answers to their questions from other sources. Caregivers’ deception of the children might be culturally acceptable from their perspective, but it was unacceptable to the children. Skovdal and Daniel (2012) argue that children’s ability for independent reflection and action in challenging social environments is key to developing strengths-based development policies and interventions that respect and build on coping strategies developed by children themselves [39].

One study that concurs with our findings explains that children in the pre-adolescent stage start to acquire more knowledge about the disease, yet they are left with many unanswered questions and misunderstandings [13]. Other studies suggest that given the number of visits they make to the hospital or clinic and the acquaintances they meet, complete unawareness at a certain age is doubtful [59]. Our data showed that the protection and prolonged silence from caregivers raised suspicions among the children. This corroborates findings from another study where the non-directness of responses from parents of HIV-infected children were observed to have an emotional effect on children [17]. Children’s inability to have an open, hopeful dialogue with their caregivers or paediatric clinicians for so long results in feelings of loneliness, sadness, and fear even after children are told [57]. The findings point to the need for a general and continuous discussion of health and illness with children from an early age followed by more specific discussions about HIV/AIDS when children are older [18].

Lastly, the older children tended to know that they were taking medicines for HIV. They increasingly wanted to be more independent and took responsibility for their medicines. The experiences of older children captured by our study are comparable to those reported in other settings. A Brazilian study on disclosure found that adolescents exhibited greater independence and assumed more responsibilities including HIV/AIDS-related care [13]. They felt that they could control information themselves. A study on HIV serostatus disclosure and lived experiences of adolescents in Uganda found that most adolescents considered information about their HIV sero-status and treatment to be a private affair [61]. Another study noted that youths discriminate and select which information they disclose about themselves and to whom [62].

Tension between caregivers and older children was reflected in the children’s assertion that they were more knowledgeable than their caregivers about issues related to their medicines and health and also had other people they could consult. The older children had less frequent communication with caregivers about their health and medicines and preferred to talk to health workers or their peers about their medicines. Evidence indicates that typically in the social world of an HIV-infected adolescent, social support networks are primarily composed of their peers [63, 64]. For adolescents with chronic conditions, peers provide support that enables them to break loose from their parents and adults in general; it is with peers that adolescents exchange ideas and feelings, and these encounters greatly contribute to identity formation [65]. For some adolescents, however, independence hid feelings of self stigma that were possibly not being addressed directly by the social support networks including the caregivers who perceived them as mature. The mature children disliked the negative messages and threats with regard to adherence to their medication. They preferred positive messages of support and encouragement to take medicines to scary messages about dying if they did not take their medicines.

This study had limitations. We were not allowed individual conversations with younger children and therefore what we know about their communication practices with them is from their caregivers’ reports. In-depth interviews were conducted with older/mature children who already knew their HIV diagnosis. In addition, the mature children preferred meeting away from their homes in places like treatment centres and clubs. We interacted little with their caregivers, so what we gathered was from their own reports.

We recognize that generalizability may be limited by the small sample size. However, we identify commonalities of children’s communication practices in relation to their caregivers with what has been documented by disclosure studies in similar resource-limited settings [13, 17] and other studies on health communication in Uganda and Kenya [50, 53]. Communication with children in these settings often is directive, rather than participatory fashion, with few opportunities for questioning, discussion, and joint decision making made available to children [17, 66]. These studies suggest that the findings may be applicable beyond the study area.

## Conclusion

While chronological age is easy and convenient to use in guidelines and research, Ugandan caregivers used their own perceptions and markers of maturity in gauging the child’s readiness for communication about HIV diagnosis and treatment. Caregiver’s perceptions of maturity, however, did not necessarily coincide with the children’s chronological age or with the children’s own perceptions of their maturity and readiness for communication. These differing perceptions of maturity and readiness resulted in tensions in communication between children and their caregivers who in most cases perceived them as too young and unready for communication about their diagnosis and treatment. Where children and caregivers perceptions of maturity and readiness coincided, there was hardly any communication between the children and their caregivers about the medications. Findings from this study suggest however that continued communication about HIV medications is critical for monitoring adherence and for supporting the children. The death of one of the adolescents in the study due to non-adherence underscores the fact that children on lifelong medication, no matter how mature they perceive themselves or are perceived by others, need to be engaged in continued communication about their medications for monitoring and support purposes. For the younger children, health care providers should encourage caregivers to recognize and respect children’s own efforts to learn about and manage (information about) their condition. Children’s questions and expressions of their feelings should be perceived as openings for communication. While a more child-centred approach might seem to undermine adult authority to assess children’s maturity, it could also be perceived as facilitating a more continuous dialogue in line with children’s own efforts to grow and live with their condition.
